# The Number of Adverse Childhood Experiences Is Associated with Emotional and Behavioral Problems among Adolescents

**DOI:** 10.3390/ijerph16132446

**Published:** 2019-07-09

**Authors:** Miriama Lackova Rebicova, Zuzana Dankulincova Veselska, Daniela Husarova, Andrea Madarasova Geckova, Jitse P. van Dijk, Sijmen A. Reijneveld

**Affiliations:** 1 Department of Health Psychology, Medical Faculty, Pavol Jozef Safarik University, Trieda SNP 1, 040 11 Kosice, Slovakia; 2 Graduate School Kosice Institute for Society and Health, Pavol Jozef Safarik University, Trieda SNP 1, 040 11 Kosice, Slovakia; 3 Olomouc University Social Health Institute, Palacky University in Olomouc, Univerzitni 22, 771 11 Olomouc, Czech Republic; 4 Department of Community & Occupational Health, University Medical Center Groningen, University of Groningen, Antonius Deusinglaan 1, 9713 AV Groningen, The Netherlands

**Keywords:** adverse childhood experiences, emotional problems, behavioral problems, adolescence

## Abstract

This study aims to examine the association of adverse childhood experiences (ACE) with emotional and behavioral problems (EBP) among adolescents and the degree to which this association is stronger for more ACE. In addition, we assessed whether socioeconomic position (SEP) modifies the association of ACE with EBP. We obtained data from 341 adolescents aged 10–16 (mean age = 13.14 years; 44.0% boys), the baseline of a cohort study. We measured EBP with the strengths and difficulties questionnaire and socioeconomic position (SEP) with self-reported financial status. We used generalized linear models to analyze the association between ACE (0 vs. 1–2 vs. 3 and more) and EBP, and the modifying effect of SEP. Adolescents with 1–2 ACE (regression coefficient: 0.19; 95%-confidence interval (CI): 0.06–0.32) and with 3 ACE and over (0.35; 0.17–0.54) reported more overall problems compared with adolescents without ACE. Moreover, adolescents with 1–2 ACE (0.16; −0.01–0.32, and 0.16; 0.03–0.29) and with 3 and over ACE (0.33; 0.10–0.56, and 0.28; 0.09–0.47) reported more emotional problems and behavioral problems, respectively. The interactions of SEP with ACE were not significant. ACE are related to EBP among adolescents, with a clear dose-response association, and this association similarly holds for all SEP categories.

## 1. Introduction

Adverse childhood experiences (ACE) relate to various negative experiences at young ages; these include abuse and/or neglect towards a child, domestic violence towards the youth’s mother, household substance abuse, household mental illness, parental separation/divorce, and a household member with a history of jail/imprisonment [1]. Their prevalence is high, e.g., Bellis et al. [2] found a lifetime cumulative prevalence of at least one ACE of 50%. Stressors such as abuse, neglect, and witnessing domestic violence are similarly common during childhood [3,4,5]. ACE can lead to signs of depression, anxiety, and personality disorder [3,6,7,8,9], showing childhood ACE to be a major public health challenge.

The experience of long-term ACE can cause serious emotional and behavioral problems (EBP) throughout the life of an individual. A dose-response has been found of ACE with various conditions, including depression, anxiety, panic reactions, hallucinations, psychosis, and suicide attempt, along with overall psychopathology, psychotropic medication use, and treatment for mental disorders. The mechanisms for these associations may involve differences in the physiological development of children or by adopting behaviors that harm their physical and mental health [6,10]. However, most of the available evidence has focused on the impact of ACE on adult health [11,12]. A recent study shows that the exposure to multiple types of ACE is associated with a higher prevalence of psychiatric disorders in adults [11]. ACE may also be expected to have effects still in adolescence [13], but evidence to support this is lacking.

Low socioeconomic position might be considered as one of the ACE itself and to a great extent causes early life stress [14,15]. It is noteworthy that experienced financial stress was found to be associated with developing mental health [16] and behavioral problems [17]. However, the role of the socioeconomic position in the relationship between ACE and EBP is still unclear.

Therefore, the aim of this study is to examine the association of adverse childhood experiences (ACE) with emotional and behavioral problems (EBP) among adolescents and the degree to which this association is stronger for more ACE. In addition, we assessed whether socioeconomic position modifies the association of ACE with EBP.

## 2. Materials and Methods

### 2.1. Sample and Procedure

We used data from the baseline wave of the Care4Youth-cohort study. We obtained participants using a two-step sampling. First, we randomly selected primary schools; these were approached from January to June 2017. Out of 11 primary schools approached, seven participated in our survey (response rate 64%). Next, parents of all pupils were asked to provide us with a signed informed consent on behalf of their children and themselves (response rate 23.4%). Questionnaires were administered by trained research assistants in the absence of teachers during regular class time. We obtained data from 341 adolescents from 5th to 9th grade aged from 10 to 16 (response rate: 94.3%, mean age: 13.14; boys: 44.0%). The study protocol was approved by the Ethics Committee of the Medical Faculty at P. J. Safarik University in Kosice (2N/2015).

### 2.2. Measures

Emotional and behavioural problems (EBP) were measured with the strengths and difficulties questionnaire (SDQ), which includes 25 items [18], of which we used the 20 difficulty items. Response categories were: not true (0), somewhat true (1), certainly true (2). The resulting score for overall difficulties can range from 0–40. In addition, we computed emotional problems (score 0–20, emotional symptoms and peer relationship problems subscales) and behavioral problems (score 0–20, conduct problems and hyperactivity/inattention subscales) [19]. A higher score indicates more problems in adolescents. Cronbach’s alpha for the whole scale was 0.78 in our sample and 0.73 and 0.71 for the internalizing and externalizing subscales, respectively.

Adverse childhood experiences were measured by the question: “Have you ever experienced any of the following serious events? (Death of a brother/sister, Death of your father/mother, Death of somebody else you love, Long or serious illness of yourself, Long or serious illness of one of your parents or of someone else close to you, Problems of one of your parents with alcohol or drugs, Repeated serious conflicts or physical fights between your parents, Separation/divorce of your parents, Separation of your parents due to work abroad). The response categories were “Yes” and “No”. We created a sum score for the number of ACE experienced, with a higher score indicating more ACE. Consequently, we categorized the number of ACE into three categories: no ACE (0), one or two ACE (1), and three or more ACE (2).

Socioeconomic position (SEP) [20] was measured using a validated tool among adolescents [21] on a 10-point scale (0—the worst, 10—the best), and the adolescents were asked to assess where they see their families on this ladder according to their financial status [22]. To illustrate what is meant, a description was provided e.g., about how much money the family had, what level of education their parents had achieved, and how profitable the work of their parents is.

### 2.3. Statistical Analyses

We first described the background characteristics of the sample, overall and by gender. Next, we assessed the association of ACE with EBP using generalized linear models adjusted for age and gender with ACE in three categories. Finally, we assessed modification of this association by the family’s SEP. Statistical analyses were performed using IBM SPSS Statistics v.20 IBM Corpotation (New York, NY, USA) for Windows.

## 3. Results

### 3.1. Descriptive Statistics

Table 1 shows the descriptive statistics of the EBP and ACE for the whole study sample and by boys and girls separately.

### 3.2. Associations between the Number of ACE and Emotional and Behavioral Problems

Table 2 presents regression coefficients (B) and 95%-confidence intervals (CI) from the generalized linear models adjusted for age and gender. Model 1 shows that adolescents with 1–2 ACE (B: 0.19; 95% CI: 0.06–0.32) and 3 or more ACE (0.35; 0.17–0.54) reported more overall difficulties in comparison with adolescents without ACE. When separately assessing emotional and behavioral problems, a similar dose-response was found with somewhat lower B coefficients. In Model 2, adolescents with a higher socioeconomic position reported fewer overall emotional (−0.05; −0.09–(−0.02)) and fewer behavioral problems (−0.05; −0.09–(−0.01)). However, the interactions of SEP with ACE were not significant (not shown in the table).

## 4. Discussion

The present study shows that ACE are associated with EBP and that the accumulation of ACE is associated with more EBP. Socioeconomic position does not significantly influence the relationship between ACE and EBP.

As suggested by our results, more ACE seems to be associated with more EBP among adolescents, thus adding to the already existing evidence on adults [6,8,9,12]. Experience of traumatic events in childhood might represent high levels of distress, which might be associated with emotional or behavioral problems [23] via enduring changes in the nervous systems [6,17,24]. In addition, it might be expected that the mentioned ACE from childhood are still present even in adolescence and have a direct and immediate influence. The deleterious effects of ACE on mental health may thus already start in adolescence.

We found the association between ACE and EBP to have a clear dose−response association, with more ACE having more pronounced influence, in line with Chapman et al. [25]. An explanation for the dose-response character of the association might be found in the resilience theory [26,27,28,29], suggesting that a lower number of ACE might be buffered by existing resilience factors, whether within the individual, family, or community [30,31]. Several simultaneously present ACE, however, might often not be resolvable by the available resources. This might lead to more EBP, thus requiring additional help from professionals from the adolescent mental health care system more frequently.

Contrary to our expectations, we found no influence of adolescents’ perceived SEP on the association between ACE and EBP. Most such studies have examined the association of SEP with ACE [9,32] or with the development of EBP [13]. Evidence on the role of SEP between ACE and EBP is scarce and inconclusive and mostly in adult populations [32,33]; our study provides additional evidence on this understudied issue. An explanation for there being no influence of adolescents’ perceived SEP on the association between ACE and EBP may be the fact that in comparison with experiencing other multiple adverse experiences in the presence of traumatic events, lower SEP might not be considered as an additional burden leading to even more pronounced EBP [34]. On the contrary, lower SEP might be expected to result in more ACE, which in turn have a detrimental influence on the development of EBP among adolescents, thus suggesting a different pathway.

This study has several strengths, the most important being that it uses validated internationally recognized instruments that have been used in various studies [35,36,37]. In addition, our study contributes to the current literature by investigating the interaction between ACE and EBP on mental health in a community-based sample of adolescents. However, this study also has some limitations. First, its response rate was rather low due to required active parental consent. However, we do not expect this to cause a major selection bias, e.g., Dent et al. [38] found no differences in mental health outcomes from studies with active and passive parental consent. Another limitation might be use of self-reported data for measuring SEP, ACE, and EBP. However, previous research has confirmed the validity of self-reported measurement of SEP [21], EBP [18], and ACE [37,39]. Finally, the cross-sectional design of this study made it impossible to formulate conclusive statements about causality.

Our study showed that ACE are associated with EBP among adolescents, with more ACE having a stronger association with EBP. These results imply a need to focus on prevention and early identification of adolescents exposed to ACE. Based on our results, implications for further research might be of interest for investigating individual ACE, as well as differentiating by the severity of the ACE. Furthermore, we in particular need longitudinal studies to assess pathways and existing mechanisms regarding the associations of socioeconomic position of the family, ACE, and EBP. Finally, research is needed on intervening in the chain of ACE towards EBP, to be able to improve future adolescent and adult mental health.

## 5. Conclusions

We found that ACE are related to EBP among adolescents and that an increasing number of ACE is associated with more EBP; SEP did not modify this association. Our results provide further evidence of associations between ACE and EBP and underscore the need for a public health and social welfare approach regarding prevention, risk reduction, and early intervention for adolescents exposed to ACE.

## Figures and Tables

**Table 1:** Descriptive statistics for age, socioeconomic position, emotional and behavioral problems, and adverse childhood experiences, overall and by gender (Slovakia 2017, 10–16 years old, *n* = 341).

	Whole Sample (*n* = 341)	Boys (*n* = 150)	Girls (*n* = 191)
Age (mean, SD)	13.14; 1.43	13.18; 1.43	13.11; 1.44
Socioeconomic position (mean, SD)	7.15; 1.56	7.21; 1.51	7.10; 1.60
EBP (mean, SD)			
Overall difficulties	11.71; 5.74	10.89; 5.62	12.36; 5.77
Emotional problems	5.07; 3.51	4.10; 3.09	5.83; 3.63
Behavioral problems	6.65; 3.43	6.88; 3.64	6.47; 3.25
ACE (*n*, %)			
No ACE	101; 31.0	44; 30.8	57; 31.1
1–2 ACE	178; 54.6	86; 60.1	92; 50.3
3 and more ACE	47; 14.4	13; 9.1	34; 18.6

SD—standard deviation; ACE—adverse childhood experiences; EBP—emotional and behavioral problems; *n*—number of respondents.

**Table 2:** Associations between the number of adverse childhood experiences (ACE) and emotional and behavioral problems, overall and separately adjusted for gender, age (Model 1), and socioeconomic position (Model 2) from generalized linear models (B coefficients/95% Wald confidence intervals) (Slovakia 2017, 10–16 years old, *n* = 341).

	Overall Difficulties		Emotional Problems		Behavioral Problems	
	Model 1 B (95% CI)	Model 2 B (95% CI)	Model 1 B (95% CI)	Model 2 B (95% CI)	Model 1 B (95% CI)	Model 2 B (95% CI)
ACE						
0	Ref.	Ref.	Ref.	Ref.	Ref.	Ref.
1–2	0.19 (0.06–0.32) **	0.18 (0.06–0.31) **	0.16 (−0.01–0.32)	0.15 (−0.01–0.31)	0.16 (0.03–0.29) *	0.15 (0.02–0.28) *
3 and more	0.35 (0.17–0.54) ***	0.30 (0.12–0.49) **	0.33 (0.10–0.56) **	0.28 (0.05–0.51) *	0.28 (0.09–0.47) **	0.23 (0.05–0.42) *
Gender						
Boys	Ref.	Ref.	Ref.	Ref.	Ref.	Ref.
Girls	0.12 (0.00–0.23) *	0.11 (0.00–0.23) *	0.31 (0.17–0.46) ***	0.32 (0.17–0.46) ***	−0.08 (−0.19–0.04)	−0.08 (−0.19–0.04)
Age	0.04 (0.00–0.08) *	0.03 (−0.01–0.07)	0.03 (−0.02–0.08)	0.02 (−0.03–0.07)	0.05 (0.00–0.09) *	0.04 (−0.00–0.08)
Socioeconomic position (0–10)		−0.05 (−0.09–(−0.02)) ***		−0.04 (−0.09–0.00)		−0.05 (−0.09–(−0.01)) *
Pseudo R^2^	0.0787	0.1048	0.0843	0.096	0.0505	0.0679

* *p* < 0.05; ** *p* < 0.01; *** *p* < 0.001.
